# Supplementary material for: Sociodemographic and clinical characteristics of people with ostomy and the adaptive domains of Roy’s theory: A cross-sectional study
Source: PLoS One. 2024 Apr 18;19(4):e0302036. doi: 10.1371/journal.pone.0302036 (PMC11025736; doi:10.1371/journal.pone.0302036)
Supplement: Attachment — Submitted filename: Response to reviews version2.docx [file pone.0302036.s001.docx]

07 march 2024

Plos One

Subject: Manuscript SOCIODEMOGRAPHIC AND CLINICAL CHARACTERISTICS OF PEOPLE WITH OSTOMY AND THE ADAPTIVE DOMAINS OF ROY'S THEORY: A cross-sectional study

- Reviewers’ and Academic editor comments for authors

Thank you very much for agreeing to consider our manuscript entitled “SOCIODEMOGRAPHIC AND CLINICAL CHARACTERISTICS OF PEOPLE WITH OSTOMY AND THE ADAPTIVE DOMAINS OF ROY'S THEORY: A cross-sectional study"

We are pleased to submit the revised version of the manuscript and we would like to express our gratitude to the reviewers and the editor for their valuable feedback, which has significantly improved the quality of the article. Reviewer 2's comments are the same as the previous review, and were already solved in the previous version. In ANNEX 1, we have provided detailed responses to each comment made by the Reviewers. All of the proposed changes have been incorporated into the revised text and highlighted in yellow.

We would be very grateful if you could consider our manuscript to be published in your journal.

Thank you for your consideration of this manuscript.

Yours sincerely,

The authors

**ANNEX 1**

| **Reviewer 1** | **Author´s response** | **Changes made to the article** | **Page/line number** |
| --- | --- | --- | --- |
| 1. If the authors have adequately addressed your comments raised in a previous round of review and you feel that this manuscript is now acceptable for publication, you may indicate that here to bypass the “Comments to the Author” section, enter your conflict of interest statement in the “Confidential to Editor” section, and submit your "Accept" recommendation. **All comments have been addressed in previous version** | Thank you for all considerations |  |  |
| **Reviewer 2** | **Author´s response** | **Changes made to the article** | **Page/line number** |
| 1. If the authors have adequately addressed your comments raised in a previous round of review and you feel that this manuscript is now acceptable for publication, you may indicate that here to bypass the “Comments to the Author” section, enter your conflict of interest statement in the “Confidential to Editor” section, and submit your "Accept" recommendation. **All comments have been addressed in previous version** | Thank you for all considerations |  |  |
| 2. Is the manuscript technically sound, and do the data support the conclusions? | The term influence written in the conclusion was replaced by association to better reflect the results |  |  |
| 3. Has the statistical analysis been performed appropriately and rigorously? | statistical analysis was adjusted. |  |  |
| **The comments below are similar to the comments requested in the previous version. Comments have been responded to and are highlighted in yellow in the revised manuscript** | | | |
| **Comment 1**  This is a single centre study – a tertiary care centre. It is therefore difficult to know how applicable these results would be to patients in different health settings. | Author’s Response comment 2:  The study was carried out in a health center that is a reference for the entire State. The State of Rio Grande do Norte has a population of more than 3.4 million inhabitants.  Due to the study design, it is possible to compare similar studies, but in fact it has limitations regarding inference for other populations. However, due to the innovative nature of the study, other similar studies may be carried out following its publication. |  |  |
| **Comment 2**  The authors do not explain how they chose the 200 participants for the study. They state that 410 patients were registered with the centre at this time period and then mention a sample of 199 eligible patients. I am not sure if they mean that 199 patients were required to obtain statistical significance, or whether there were only 199/410 patients who were eligible. Furthermore, it is not clear if selected patients were consecutive, or randomly chosen, or selectively chosen. This needs to be clarified, so that the results can be interpreted in the light of the included patients. | **Author’s Response comment 2:**  the sample calculation formula and the method for selecting those surveyed were added | When considering a population (N) of 410 patients registered at the center in 2017, after performing the sample calculation, with confidence level of 95.0% and margin of error of 5.0% (n=N.Z^2^.p.(1-p) / Z^2^.p.(1-p) + e^2^.N-1 ) a sample (n) of 199 individuals were required to obtain statistical significance. From Monday to Friday the researchers went to the center until they reached a sample of 200 people (consecutive chosen). | Page 5 |
| **Comment 3**  In the section on “instruments and variables” – last paragraph. The final two sentences do not make sense. The penultimate sentence on the scoring system is very confusing and makes no sense. This needs to be either rewritten completely or a table with the scoring system should be inserted. | **Author’s Response comment 3:**  The sentence was improved and we added a table with the instrument's scores |  | Page 5 |
| **Comment 4**  Results section – this needs to be improved with the addition of a new Table 1 which gives the demographic data on the included patients. This would be the baseline against which the following results could be analysed. It is essential to set out how many male/female patients, ages, etc. If a new Table 1 is inserted, then the numbers of the other tables will all be moved on and this should be altered in the text as well as on the legends. | **Author’s Response comment 4:**  A table with sociodemographic and clinical characterization variables was added on page 7 |  | Page 7. |
| **Comment 5**  In the discussion the authors state that lack of family support links with poor scores across several domains. However, the results are only for lack of a partner, rather than all family support. This is a subtle difference as many family members/friends can provide support whether the patient has a partner or not. | **Author’s Response comment 5:**  The excerpt discusses the findings of other authors (Gautam S, Poudel A. Efeito do gênero no ajustamento psicossocial de sobreviventes de câncer colorretal com ostomia. J Gastrointest Oncol. Dezembro de 2016;7(6):938-945. doi: 10.21037/jgo.2016.09.02. PMID: 28078117; PMCID: PMC5177570) on the results of low psychosocial adjustment in men compared to women. In this study, this result occurred due to the perceived lack of family support, total dependence on others to care for the ostomy and the professional situation. The comparison made with our work is due to the low adjustment among men. | Paragraphs were added to address this result of people living with and without a partner. The importance of social support was also mentioned, which does not only come from partners, but from family, friends and professionals | Page 10 and 11. |
| **Comment 6**  the sentence on Study Limitations does not make sense. There are many limitations to this study and at least one paragraph should be devoted to describing them in more detail. | Limitations have been rewritten | Limitations regarding generalization and other possibilities for analysis were explored. | Page 14 |
| **Reviewer 2 - TYPOGRAPHICAL AND GRAMMATICAL ERRORS** | | | **Author´s response** |
| Page 1 TITLE – There are two : after “Theory” – please delete one of them. ABSTRACT -In the sentence regarding Results- Please change from “significant associations in the male sex” to “significant associations with male sex” INTRODUCTION Page 2 – paragraph 2 – there is a line ____________ before the first word in this paragraph. Please delete. Page 2 - Paragraph 3 – line 3 – change “for eliminations” to “for elimination” Page 2 – Paragraph 3 – line 5 – change “elimination” to “evacuation” Page 2 – Paragraph 3 – line 5 – remove the commas after “bag” and after “social events” Page 2 – Paragraph 4 – Line 1 – should start with “A study” not just “Study” Page 2 – Paragraph 5 – line 3 – Pleas delete “for” before “health professionals” Page 2 – Paragraph 5 – line 5 – please change “this characteristics” to “the characteristics” Page 2 – Paragraph 5 – line 7 – please change “support assistance” to “provide assistance” MATERIALS AND METHODS Page 3 – The title of the section should be “Materials and Methods” not “Material and Methods” Ethical Considerations – second paragraph – please change “clarifications” to “information” Ethical Considerations – second paragraph – line 2 – please change “the written signature of the term” to “a written signature” Population and Sample section – line 2 please put a : rather than ; after “stoma” Statistical analysis section – last paragraph – line 2 – change “are independent” to “of the independent” Statistical analysis section – last paragraph – line 4 - change "by the absence” to “because of the absence” RESULTS Tabel 1 – there is a spelling mistake in the left column – It should be “Age Range” not “Age ranger” In the results section – the penultimate paragraph – should be changed from- “all of them.. they following stood out” to- “all of them. The following stood out” DISUCSSION Paragraph 1 - Line 1 – change “male” to “males” Paragraph 2 – Line 4 – change “because lack of” to “because of lack of “ Paragraph 6 – Line 1 – please change “see if elderly people have better results” to “elderly people had better results” Paragraph 7- Line 2 – please delete Wince” Paragraph 7 – Line 3 – please change “give importance” to “give due consideration” Paragraph 8 – Line 2 – please change “the stoma construction. Colostomy people fear” to “stoma construction. People with an ostomy fear” Paragraph 8 – Line 3 – please change “you’re” to “they are” Paragraph 8 – Line 4 – please change “he fear of leaks” to “the fear of leaks” Paragraph 9 – Line 3 – please change “favors” to “supports” Paragraph 9 – Line 4 - please change “with ostomy” to “with an ostomy” Paragraph 11 – Line 2 – please change “ as they do not work and have pensions” to “as those who do not work or have pensions” Paragraph 11 – Line 3 – please change “but that become” to “but that is” Paragraph 12 – Line 4 - please change “population, however many of them are unaware” to “However, many of the population are unaware of these opportunities” Paragraph 14 – Last line – please change “those aged three years or older” to “those persisting for three years or more” Paragraph 18 - line 1 – please change “scholarship” to “device” Paragraph 19 – Line 2 – please change "inputs” to “devices” and change “scholarship” to “device Paragraph 20 – Line 1 – please change “presented” to “developed” Paragraph 20 – line 2 - please change “all of them, in addition” to “all of them. In addition” Paragraph 22 – line 2 - please change “concluded, as people” to “concluded that people” Paragraph 22- line 4 – please change “presented” to “developed” Paragraph 22 – line 4-5 - please change “many were not attended to or received guidance for the care of injuries” to “many did not receive care or support with these problems” Paragraph 24 – Line 4 – please change “not acceptable, but” to “unchangeable and” Paragraph 24 – the last sentence does not make sense. It should either be explain clearly or removed. Paragraph 25 – Line 1 – please delete “the” before “younger” Paragraph 26 – the first sentence does not make sense - do the authors mean that the length of time that a stoma has been present does not affect self-care? Last paragraph of results section – please change – “between characteristics sociodemographic and clinical and the adaptation” to “between sociodemographic and clinical characteristics and adaptation” CONCLUSION- Paragraph 1 – Line 4- please change “one year had” to “one year also had” Paragraph 1 – Line 4 - please change "associations to” to “associations with” Paragraph 1 – last line - please change “with all” to “across all” Paragraph 2 – line 2 – please change “subsidies” to “information” Paragraph 3 – Line 1 - please change “The study” to “This study” | | | All corrections were made to the text and are highlighted in yellow |
